# MARCKS promotes invasion and is associated with biochemical recurrence in prostate cancer

**DOI:** 10.18632/oncotarget.18894

**Published:** 2017-06-30

**Authors:** Emma Dorris, Amanda O'Neill, Karen Hanrahan, Ann Treacy, R. William Watson

**Affiliations:** ^1^ UCD School of Medicine, Conway Institute for Biomedical and Biomolecular Sciences, University College Dublin, Belfield, Dublin 4, Ireland; ^2^ Pathology Department, Mater Private Hospital, Dublin 7, Ireland

**Keywords:** prostate cancer, biochemical recurrence, MARCKS, invasion, migration

## Abstract

**Background:**

Overtreatment of low-grade prostate cancer is a recognised problem for clinicians and patients. However, under-treatment runs the risk of missing the opportunity for cure in those who could benefit. Identification of new biomarkers of disease progression, including metastases, is required to better stratify and appropriately treat these patients. The ability to predict if prostate cancer will recur is an important clinical question that would impact treatment options for patients. Studies in other cancers have associated MARCKS with metastasis.

**Methods:**

Tissue microarrays of local prostatectomy samples from a cohort of biochemical recurrent and non-biochemical recurrent tumours were assayed for MARCKS protein expression. Prostate cancer cell lines were transfected with siRNA targeting MARCKS or a control and functional endpoints of migration, invasion, proliferation, viability and apoptosis were measured. Actin was visualised by fluorescent microscopy and evidence of a cadherin switch and activation of the AKT pathway were assayed.

**Results:**

MARCKS was upregulated in biochemical recurrent patients compared to non-biochemical recurrent. Knockdown of MARCKS reduced migration and invasion of prostate cancer cells, reduced MMP9 mRNA expression, as well as decreasing cell spreading and increased cell:cell adhesion in prostate cancer cell colonies. Knockdown of MARCKS had no effect on proliferation, viability or apoptosis of the prostate cancer cells.

**Conclusions:**

In conclusion, MARCKS promotes migration and invasion and is associated with biochemical recurrence in localised prostate cancer tumours. The mechanisms by which this occurs have yet to be fully elucidated but lack of a cadherin switch indicates it is not via epithelial-to-mesenchymal transition. Actin rearrangement indicates that MARCKS promotes invasion through regulating the architecture of the cell.

## INTRODUCTION

Prostate cancer accounted for approximately 15% of new cancer cases diagnosed in men worldwide in 2012. Age adjust incidence rates of prostate cancer have increased dramatically, with incidence of prostate cancer doubling in Ireland between 1995-2007 [1]. The rise in incidence is largely due to increased screening for prostate specific antigen (PSA), which can lead to the detection of small or indolent cancers that would otherwise remain undetected and which may or may not develop into high grade disease. Early metastatic disease is difficult to detect. The ability to predict if a cancer will metastasise is an important clinical question that would impact treatment options for patients.

Within 10 years of radical prostatectomy, approximately one third of men have detectable PSA increases [2–6]. This rise in serum PSA following definitive therapy is termed biochemical recurrence (BCR) and is often the first evidence of disease recurrence. BCR can occur in isolation, without objective findings and the clinical landscape of patients with BCR can be extremely variable [7]. Understanding the biology behind progression to BCR prostate cancer will help elucidate the mechanisms of failure to primary treatment and advance our understanding of disease progression.

Radical prostatectomy is the primary treatment for localised medium and high grade prostate cancer. Disease recurrence is assessed based on periodic measurement of PSA. A detectable rising PSA after radical prostatectomy is considered BCR [8] and is indicative of disease progression, even in the absence of clinical symptoms [3]. Identification of new markers of disease progression, including metastases, is required to better stratify and appropriately treat patients. The ability to predict if a cancer will metastasise is an important clinical question that would impact treatment options for patients. MARCKS (Myristoylated Alanine-Rich Protein Kinase C Substrate) is a protein involved in regulating the architecture of the cell [9]. In order to metastasize, the cell must be able to change its shape numerous times to migrate out of the tissue, travel through the lymph or blood stream and invade at a new location. Thus, MARCKS could be an interesting biomarker and target for cancer metastasis. MARCKS interacts with the plasma membrane and crosslinks filamentous- (F-) Actin [9]. Upon phosphorylation by protein kinase C (PKC) or binding to calmodulin, MARCKS dissociates from the plasma membrane. MARCKS interacts with PKC and calmodulin in a mutually exclusive manner and can therefore regulate crosstalk between the two signalling pathways [10].

The role of MARCKS in prostate cancer has not previously been studied but has been associated with metastasis and disease progression in lung and colon cancer [11, 12]. In other cancers MARCKS has been described as both tumour suppressive and oncogenic, dependent upon tumour type [11, 13–16]. MARCKS is frequently inactivated in colorectal cancer and this inactivation is associated with adverse outcomes [13]. Conversely, MARCKS is elevated in highly invasive lung cancer [11] and associated with invasion in leukemia cells [17]. Furthermore, experimental treatment with a peptide that inhibits MARCKS function reduced lung cancer metastasis *in vivo* [11].

In this study we have characterised a functional role for MARCKS in prostate cancer by investigating its role in proliferation, viability, apoptosis, migration and invasion. We provide evidence that MARCKS promotes migration and invasion in prostate cancer cells and that this transfers clinically, as MARCKS is upregulated in BCR compared to non-BCR men with prostate cancer. This does not seem to occur directly via epithelial-to-mesenchymal transition (EMT) as demonstrated by lack of a cadherin-switch.

## RESULTS

**Figure 1:**
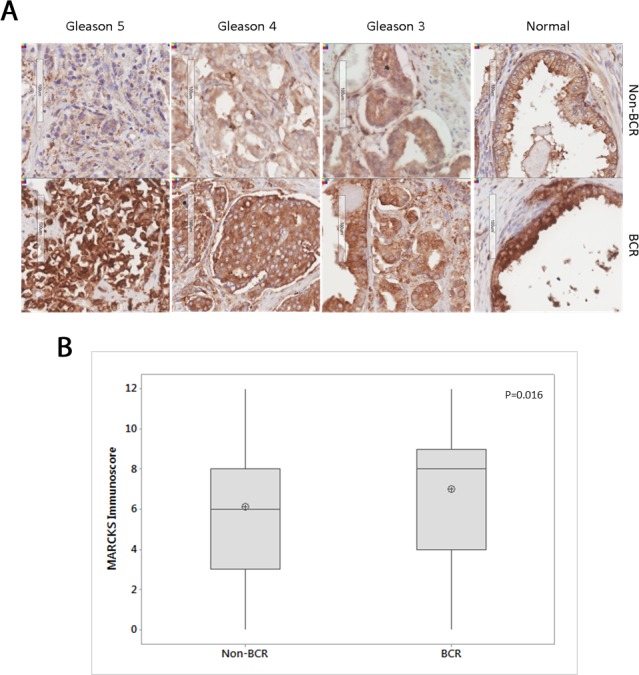
MARCKS staining in non-BCR and BCR cohorts **(A)** Representative IHC staining of MARCKS protein in different Gleason grades in non-BCR (n=36) and BCR (n=33) cohorts. **(B)** Boxplot of MARCKS staining immunoscore between non-BCR and BCR cohorts. Central line in boxplot represents median, circle represents mean. p=0.016 Pearson's Chi-square test.

To identify MARCKS expression in prostate tissue, immunohistochemical staining was performed on tissue microarray (TMA) slides containing prostatectomy samples from 69 patients. Patients are all from the Irish Prostate Cancer Research Consortium Bioresource and cohorts consisted of biochemical recurrence (BCR) patients n=33 (n=231 cores) and non-BCR patients n=36 (n=283 cores). Clinical data for both cohorts is outlined in Table 1. Cores were scored by an independent pathologist and the immunoscore was calculated using the Loda method [18]. Overall, MARCKS staining is increased in normal tissue compared to Gleason grades 3, 4, 5 (ANOVA with Tukey's HSD post-hoc correction, p=0.000). This does not differ by BCR status. MARCKS was increased in BCR cohort compared to non-BCR (Pearson's Chi-square p=0.016, Figure 1).

**Table 1:** Clinical features of prostate cancer patient cohorts

Clinical Features	Non-BCR		BCR	
	*Median*	*Range*	*Median*	*Range*
**Age at Time of Surgery**	64	(46-69)	63	(42-73)
**PSA (ng/ml)**	7.75	(1.9-17)	7.67	(1-18.5)
**Biochemical progression free (months)**	83	(27-109)	n/a	n/a
**Time to Biochemical recurrence (months)**	n/a	n/a	11	(2-72)
	***n=***	***% Cohort***	***n=***	***% Cohort***
**RRP Gleason Score**				
**6**	8	22.22	11	33.33
**7**	20	55.56	15	45.45
**8**	6	16.67	6	18.18
**9**	2	5.56	1	3.03
**Organ Confined T2a/b**				
**Negative**	32	88.89	29	87.88
**Positive**	4	11.11	4	12.12
**Organ Confined T2c**				
**Negative**	17	47.22	25	75.76
**Positive**	19	52.78	8	24.24
**Extracapsular Extension (T3a)**				
**Negative**	24	66.67	17	51.52
**Positive**	12	33.33	16	48.48
**Seminal Vesical Invasion**				
**Negative**	35	97.22	28	84.85
**Positive**	1	2.78	5	15.15
**Lymphnode Invasion**				
**Negative**	5	13.89	3	9.09
**Positive**	1	2.78	2	6.06
**Not Performed**	30	83.33	28	84.85
**Mortality**				
**No**	35	97.22	25	75.76
**PCa**	0	0.00	1	3.03
**Other Cause Mortality**	0	0.00	0	0.00
**No Data**	1	2.78	7	21.21
**Mets**				
**No**	35	97.22	21	63.64
**Yes**	0	0.00	4	12.12
**No Data**	1	2.78	8	24.24

**Figure 2:**
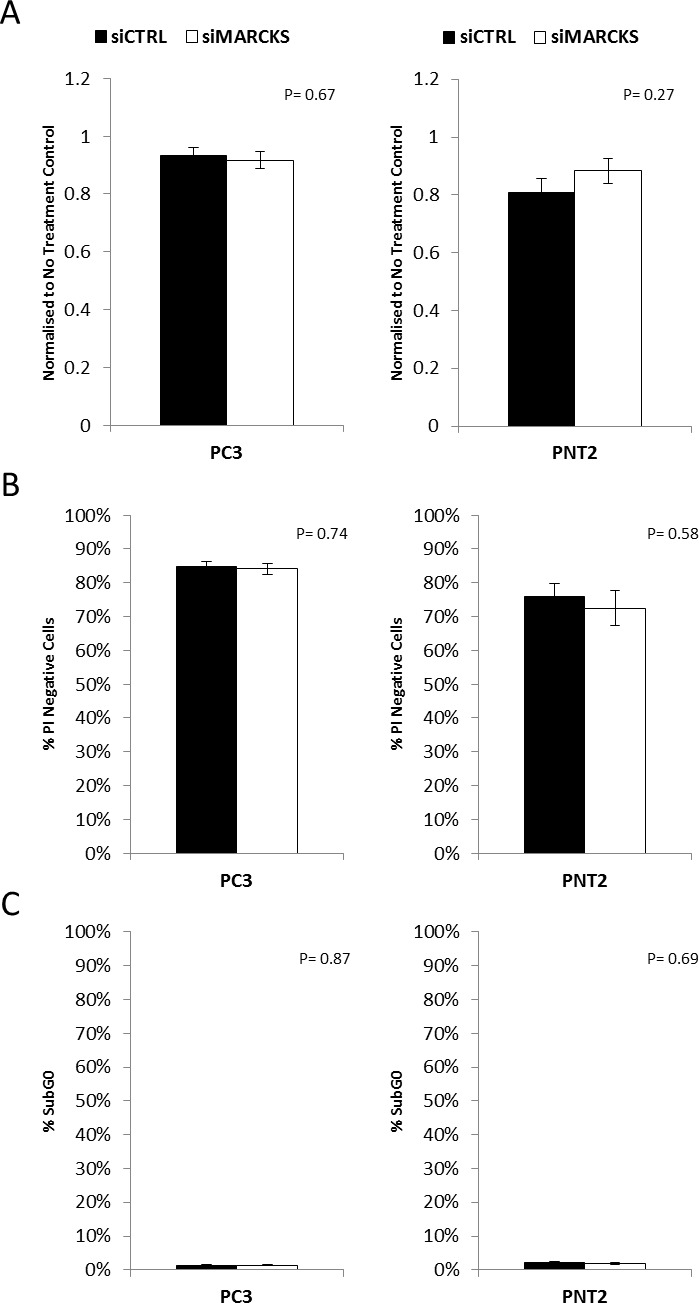
MARCKS does not play a role in prostate cancer cell viability MARCKS knockdown did not alter the **(A)** proliferation **(B)** viability or **(C)** basal apoptosis of prostate cancer cells. Error bars +/− SEM. P-value calculated using Student's T-Test n=3 independent experiments.

To determine the functional effect of MARCKS in prostate cancer, a siRNA-mediated knockdown of MARCKS was used. All experiments were performed in the prostate cancer cell lines PC3, a highly invasive cell line, and PNT2, a transformed normal prostate cell line. PC3 is tumorigenic in mice whereas PNT2 is non-tumorigenic in nude mice [19, 20] Knockdown of MARCKS did not have any effect on cellular proliferation of prostate cancer cell lines (Figure 2). Furthermore, the viability and level of basal apoptosis was not significantly affected by knockdown of MARCKS (Figure 2).

**Figure 3:**
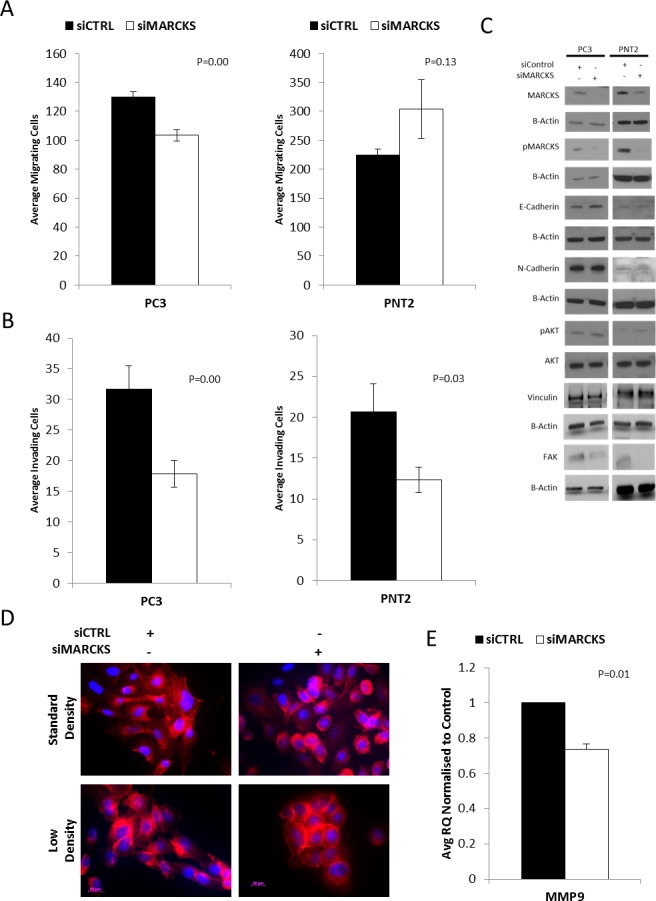
MARCKS promotes invasion and is associated with altered actin patterning **(A)** MARCKS knockdown reduces migration of the aggressive prostate cancer cell line PC3 (P≤0.001) but not the well-differentiatedPNT2 cell line. **(B)** MARCKS knockdown decreases invasion in both the PC3 (P≤0.001) and PNT2 (P≤0.05) cell line. **(C)** Representative (n=3 individual experiments) cropped western blots showing MARCKS knockdown. There is no evidence of a cadherin switch, decrease in Vinculin or a perturbance in the AKT pathway in response to MARCKS knockdown. There is decrease in FAK in the siMARCKS treated cells. **(D)** Immunofluorescent staining of actin (red) in response to MARCKS knockdown demonstrates less surface protrusions and cell spreading in PC3 cells. PC3 cell colonies treated with siMARCKS are more compact with more cell-cell adhesion than those treated with control (non-targeting) siRNA. **(E)** *MMP9* gene expression is decreased in PC3 cells following MARCKS knockdown. Error bars +/− SEM. P-value calculated using Student's T-Test, at least n=3 individual experiments except for the FAK PNT-2 western which is an n=1.

In the aggressive cell line PC3, knockdown of MARCKS significantly decreased migration of cells through transwell chambers (p=0.00) compared to cells transfected with a non-targeting siRNA (Figure 3A). There was no significant difference observed in the migrative capacity of PNT2 following MARCKS knockdown. The invasive capacity of the cells was significantly decreased following MARCKS knockdown in both PC3 (p=0.00) and PNT2 (p=0.03) cells (Figure 3B). PNT2 had a higher number of migrating cells per field compared to PC3 cells and a lower number of invading cells per field. The percentage of invading cells compared to migrating cells was 9% in PNT2 compared to 24% in PC3 (siControl group), reflective of the more aggressive nature of PC3 cells. PC3 cells also had higher levels of phosphoMARCKS relative to total MARCKS compared to PNT2 cells (western blot densitometry [21] average normalised to endogenous controls PC3 cells 2.62 +/− 1.65 pixels compared to PNT2 cells 0.52 +/− 0.17 pixels), consistent with previous observations that phosphoMARCKS is associated with more aggressive cancer cells [14, 22]. The gene expression of matrix metalloproteinases (MMPs) was measured in PC3 cells following MARCKS knockdown. *MMP1* and *3* showed no significant difference (p=0.26 and p=0.56 respectively). However, *MMP9* gene expression was significantly decreased upon MARCKS knockdown (p=0.01). This further demonstrates the ability of MARCKS to regulate invasion. To elucidate the mechanism behind this increased invasive capacity, a number of proteins were assessed by western blotting following MARCKS knockdown or non-targeting control.

Cadherin switching has previously been reported as a mechanism of prostate cancer progression [23]. Cadherin switching is a signature event in the epithelial-to-mesenchymal transition, a well-documented regulatory network involved in invasion of tumour cells [24]. Levels of E-Cadherin (CDH1) and N-Cadherin (CDH2) were measured in PC3 and PNT2 cells transfected with siRNA targeting MARCKS or non-targeting siRNA. The levels of N-Cadherin were readily detectable in PC3 cells but very low in PNT2 cells, consistent with previously reported studies that N-cadherin is not expressed in well differentiated prostate tissue but expression increases as differentiation decreases [23]. There was no evidence of a cadherin switch following MARCKS knockdown, indicating that an epithelial-to-mesenchymal transcriptional pathway is not induced by MARCKS knockdown (Figure 3C).

MARCKS is associated with the PI3K/AKT pathway via its ability to interact with and sequester phosphatidylinositol 4,5-bisphosphate (PIP2) [25, 26]. MARCKS knockdown has previously been shown to decrease the levels of phosphorylated AKT in lung cancer cells [11]. However, we observed no difference in the levels of AKT in response to MARCKS knockdown in either of the prostate cancer cell line models (Figure 3C).

The main function of MARCKS is the regulation of actin [9, 10, 27]. Therefore, we observed the actin architecture by immunofluorescent staining following MARCKS knockdown (Figure 3D). Following MARCKS knockdown, the cells appear rounder with a reduced level of surface protrusions and a reduction in cell spreading. Cells were also seeded at low density to observe colony formation. Based on the actin staining, cell colonies with MARCKS knockdown appear to exhibit more cell-cell adhesion and were more compact than control colonies. As Focal adhesions anchor cells and regulate migration we next assessed focal adhesion kinase (FAK) and vinculin key regulators of focal adhesions [28]. Figure 3C demonstrates no change in vinculin but a down regulation of FAK in the MARCKS knockdown PC3 and PNT2 cells.

## DISCUSSION

The results of this study have identified for the first time an increased expression of MARCKS protein in clinical samples of prostate cancer from BCR patients compared to non-BCR patients. This observation prompted the characterisation of MARCKS function in prostate cancer cell lines. Using siRNA-mediated knockdown of MARCKS, prostate cancer cell lines showed reduced invasion. Analysis of MMPs demonstrated that this reduced invasion may in part be mediated by a reduction of *MMP9*. Basal rates of proliferation, viability and apoptosis were not affected by MARCKS knockdown. MARCKS has previously been reported to promote invasion in lung cancer via an association with the PI3 Kinase/AKT pathway [11]. However, in prostate cancer cells there is no evidence for altered activation of AKT in response to MARCKS knockdown.

EMT is strongly linked with disease progression in prostate cancer [23, 29–33]. A cadherin switch between E-Cadherin and N-Cadherin is a classic marker for EMT [34]. E-Cadherin is a cell surface protein that binds cells together in normal epithelial cells. N-Cadherin is a cell-surface protein usually expressed in neural cells but upon induction of EMT it is induced and associated with the progression from well differentiated adenoma to invasive carcinoma [23]. There was no evidence of a cadherin switch in response to MARCKS knockdown. The levels of N-Cadherin were extremely low in the PNT2 cell line, reflecting the more differentiated phenotype of the cell line compared to the aggressive PC3 cell line, in which N-Cadherin was readily detectable. The promotion of invasion by MARCKS does not appear to be mediated by EMT in prostate cancer.

A prerequisite for a complete malignant phenotype is the acquisition of the capacity to migrate and to invade into a tissue. In order to invade and metastasize, a cell must undergo a number of morphological changes. These morphological changes are driven, at least partially, by remodelling the actin cytoskeleton. The role of MARCKS in promoting metastases in colon and lung cancer has recently been demonstrated [11, 12]. MARCKS is well characterised as a regulator of actin cytoskeleton remodelling [9, 27, 35]. MARCKS cross-links actin filament and this activity is inhibited by protein kinase C (PKC)-mediated phosphorylation or by binding to calcium-calmodulin [9]. Here, we have demonstrated that knockdown of MARCKS alters actin arrangement in prostate cancer cells. Reduced MARCKS reduces surface protrusions and the extent of cell spreading. The precise adhesion molecules involved in MARCKS-induced actin rearrangement of prostate cancer cells have yet to be elucidated but we have demonstrated that MARCKS knockdown is associated with no change in vinculin expression which is supported by the fact that vinculin is not found in dynamic adhesions which contain alpha3integrins and MARCKS [36]. We did however demonstrated that FAK is down regulated in the MARCKS knockdown cells which is a primary regulator of focal adhesion signalling and loss of FAK drives more stable focal adhesion and lose of migration [28]. Further work around the mechanisms by which MARCKS regulates FAK is ongoing. Furthermore, when seeded at low density to observe colony formation, colonies with MARCKS knockdown exhibited more cell-cell adhesion and tightly packed colonies. These results are consistent with those observed in lung cancer cells treated with a MARCKS inhibitor [11]. Thus, in prostate cancer, MARCKS promotes invasion through its regulation of the cytoskeleton.

## CONCLUSIONS

In summary, our results show a role for MARCKS in promoting invasion in prostate cancer. Consistent with this, we have demonstrated an association of MARCKS with BCR in prostate cancer clinical samples. The characterisation of the role of MARCKS in prostate cancer, as outlined here, provides a basis for future studies to elucidate the precise mechanism behind MARCKS-induced invasion and whether its inhibition can be used therapeutically to prevent prostate cancer disease progression.

## MATERIALS AND METHODS

### Ethics statement

Investigation has been conducted in accordance with the ethical standards and according to the Declaration of Helsinki and according to national and international guidelines and has been approved by the authors’ institutional review board. Biological material used in this study was from the Irish Prostate Cancer Research Consortium Bioresource. Samples were collected using the federated collection model. The use of tissue samples was approved by the Ethics Committee of the Mater Misericordiae University Hospital and all patients gave written consent.

### Tissue microarray

Tissue microarray (TMA) contained 1mm cores from formalin fixed paraffin embedded radical prostatectomy (FFPE) tissues from 69 patients from the Irish Prostate Cancer Research Consortium Bioresource. TMAs contained three cores from each grade of tumour, where possible. “Normal” tissue from each FFPE block was also included in triplicate. The total number of patient cores was 514 across 8 slides. Liver, colon and tonsil cores were included in the TMA to act as controls. A random layout of cores was used to prevent reading bias. All patients were from an Irish population with a median age of 63 and pre-operative serum PSA of 7.67 see full clinical details are contained in Table 1. Patients were selected and matched into two cohorts based on a five-year follow-up for biochemical recurrence: BCR patients n=33 (n=231 cores), non-BCR patients n=36 (n=283 cores).

BCR was defined using the Stephenson definition of 2 follow up PSA values of greater than 0.4ng/ml at least 1 month following surgery [37].

### Immunohistochemistry

IHC staining was performed using a microwave-induced antigen retrieval method as previously described [38]. De-waxed sections were immersed in a citric acid buffer (0.01 M, pH 6.0), placed in a 800-W microwave oven at full power for 15 min. Using a standard avidin-biotin complex method (Vector Laboratories, Inc.), the sections were incubated with monoclonal rabbit anti-MARCKS (AbCam, 1:300 dilution [39]) at 4°C overnight. The color reaction product was obtained with DAB and counterstained with Hematoxylin. Prior to this study, the MARCKS antibody was subjected to Western blot analysis using prostate cell lines which confirmed specificity for MARCKS. Immunoexpression of MARCKS was performed by an independent pathologist. Cores were given an intensity score (0-3, with 0 being no expression, 1 mild, 2 moderate and 3 intense) and a percentage of positive cells. These were used to calculate the immunoscore based on the Loda method [18]. The percentage of positive cells was given the following weightings: 0%- 0, 1-9%-1, 10-39%-2, 40-69%-3, and 70-100%-4. The intensity score was then multiplied by the percentage score to obtain the final immunoscore. Pearson Chi-Squared tests were performed in Minitab statistical software to test for association between MARCKS immunoscore and biochemical recurrence. ANOVA with Tukey's honest significant difference post-hoc test was performed in Minitab to test for differences between Gleason grades.

### Cell culture

PNT2 cells were purchased from the European Collection of Cell Cultures (ECACC) and PC3 cells were purchased from the American Type Culture Collection (ATCC). PNT2 cells were used within 6 months of receipt from ECACC and PC3 cells were authenticated (DDC Medical Human Cell Line Authentication Service) prior to use. Both cell lines were maintained in RPMI-1640 medium supplemented with 10% Fetal Bovine Serum (FBS), 50 U/ml penicillin/50 μg/ml streptomycin and 2 mM L-glutamine (Invitrogen).

### Quantification of apoptosis and viability

Apoptotic events were described as a percentage of total events with hypodiploid DNA assessed by cellular incorporation of the DNA-intercalating agent propidium iodide (PI) upon membrane permeabilisation as previously described [40]. Briefly, cells were harvested by trypsinisation, permeabilised with a hypotonic fluorochrome solution (50 mg/ml PI, 3.4 mM sodium citrate, 1 mM Tris, 0.1 mM EDTA, and 0.1% Triton X-100) and incubated on ice for 10 minutes prior to analysis. PI viability assays were performed to distinguish between the intact membranes of normal and apoptotic cells and the disrupted membranes of necrotic cells. Cells were harvested by trypsinisation prior to addition of a PI solution without Triton X (50 μg/ml PI, 3.4 mM sodium citrate, 1 mM Tris, 0.1 mM EDTA) and incubated in the dark at 4°C for 15 minutes prior to analysis. Samples were run on an Accuri C6 Flow Cytometer (BD Biosciences). Ten thousand (apoptotic) or twenty thousand (viability) events were gated on PI intensity and analysed using CFlow Plus Software (BD Biosciences).

### Cell proliferation (MTT) assay

Cells were seeded at 3.75×10^4^ cells per ml into Nunclon Δ 96-well plates (Nunc) at 200μl per well and incubated under standard conditions for 48 hours. Cells were transfected with siRNA and incubated for 48 hours. The Cell Proliferation Kit I (MTT) (Roche) was employed to measure the growth rates of cells. A control with no treatment added was included on all plates and used to normalise results.

### Migration and invasion transwell assay

Cells were seeded into 6-well plates at 250,000 cells per well and incubated for 24 hours. Cells were transfected with siRNA and incubated for 24 hours. Cells were trypsinised and each well resuspended in 1ml of serum-free media. 300μl cells were added to the chambers of uncoated (migration) or matrigel-coated (invasion) transwell chambers sitting in 24-well plates. 500μl of full media was added per lower well and plates incubated for 48 hours. Cells were removed from the upper chamber using cotton swabs dampened in PBS and chambers were stained in 0.25% crystal violet. Chambers were divided into quadrants, each quadrant imaged at 20X and the number of cells counted per image. All chambers were set up in triplicate.

### Fluorescent microscopy

Cells were grown (37,500 (standard density) per well or 11,250 per well (low density)) and treated in Lab-Tek™ 8-well chamber slides (Nunc), fixed in 4% paraformaldehyde solution (150μl/well) and permeabilised with 0.5% Triton X solution. Actin was stained with Phalloidin-TRITC (Sigma) and Fluroshield™ with DAPI (Sigma) was added to each slide and a coverslip (#1.5) was added and sealed with a clear nail varnish top coat. Slides were mounted onto an Epi-fluorescence microscope. Images were captured at 40x magnification. Scans were performed at 1 μm interval depths through the fixed cells, and single or merged images are presented as XY single planes.

### Western blotting

Whole cell lysates were extracted and Western Blotting performed as previously described [40]. Briefly, Cells were washed in cold PBS then resuspended in NP-40, Tris 10 mM pH 8.0, 60 mM KCl, 1 mM EDTA pH 8.0, 1.0 mM DTT, 10 μl/ml Protease Inhibitor Cocktail (Sigma P8340) and Phosphatase Inhibitor Cocktail (Sigma P0044), and 10 mM PMSF. Samples were then placed on ice for 10 mins and the cell lysate collected after centrifugation (13000 rpm 10 mins at 4°C). Total cellular protein was determined by means of the Bradford Assay Protein Detection Kit (Bio-Rad). Equal amounts of protein (50 μg) were subjected to SDS polyacrylamide gel electrophoresis on 4-15% gels before being trans-blotted onto Immobilin P (Millipore) membranes. Membranes were blocked in 5% BSA prior to overnight incubation at 4°C in primary antibody. Membranes were washed in TBS-T then incubated with the appropriate horseradish peroxidase-conjugated secondary antibodies (Cell Signaling Technology (CST)). Primary antibodies were as follows: anti-MARCKS (1/5000, AbCam [13]), anti-phosphoMARCKS (1/500, CST), anti-E-Cadherin (1/1000, BD Trans [41]), anti-N-Cadherin (1/2000, BD Trans [42]), anti-AKT (1/1000, CST [43]), anti-phosphoAKT (1/1000, CST [43]), Vinculin (1/1000 Sigma V9131), anti-FAK (1/500 Transduction Laboratories F15020) and anti-β-Actin (1/5000, Sigma [44]).

### Gene expression analysis

Macherey Nagel NucleoSpin™ miRNA extraction kit was used for RNA extraction, as previously described [45]. RNA was quantitated via Nanodrop 2000 spectrophotometry. The High Capacity cDNA Reverse Transcription Kit (Applied Biosystems) was used to convert total RNA to single stranded cDNA using 200ng RNA per sample. Predesigned RT-PCR TaqMan® assays Hs00957562_m1 (MMP9), Hs00968305_m1 (MMP3), Hs00899658_m1 (MMP1) with 18S (4310893E) as an endogenous control. A non-template control was included for all assays. Biological triplicates of PC3 cells lines treated with siControl or siMARCKS were averaged and displayed normalised to siControl.

### Statistical analysis

Statistical analysis was carried out using two-sample unequal variance Student t-tests. Results were considered statistically significant where p < 0.05 and results are expressed as mean ± the standard error.

## Abbreviations

PSA: Prostate Specific Antigen
BCR: Biochemical Recurrence
MARCKS: Myristoylated Alanine-Rich Protein Kinase C Substrate
PKC: Protein Kinase C
EMT: Epithelial-to-Mesenchymal Transition
TMA: Tissue Microarray
FFPE: Formalin Fixed Paraffin Embedded
IHC: Immunohistochemistry
CDH1: E-Cadherin
CDH2: N-Cadherin
PIP2: Phosphatidylinositol 4,5-bisphosphate
MMP: Matrix Metalloproteinase
FAK: Focal adhesion kinase
